# The Condition of Midwives

**Published:** 1887-08-20

**Authors:** 


### THE CONDITION OF MIDWIVES.

Sir,—A few facts in accordance with Mrs. Bedingfeld's paper may perhaps interest some of your readers, as many people, I find, are quite unaware that the majority of women of the working-class depend entirely upon the unskilled attendance of a very low and ignorant class of midwives. In this North-country town where I am living it is the exception when a doctor is called in to any ordinary case. The midwives are women of the lowest grade, who possess, no doubt, much natural shrewdness, but who are ignorant and prejudiced to a degree almost inconceivable. One of the doctors told me that he had endeavoured to instruct them, but had given up the attempt as a hopeless task, as he had found it impossible to make them understand even the ordinary means of arresting hæmorrhage.

Of course drunkenness among them is common. Age and its infirmities, if a midwife can walk to her patient's house, do not appear to prevent her following her calling. I know one over eighty years of age, who is childish, deaf, and lame, and she still continues to go out to midwifery cases. They use disgusting and absurd remedies for ailments that are often only the consequence of improper food or want of cleanliness. When not past work they often undertake washing, besides midwifery and nursing, as they are very industrious women; but their varied and laborious employments are apt to prevent them paying proper attention to their patients, and there is much suffering from neglect. I do not speak of the gross cases where illness and death are the consequences of the incompetence of the midwife. E. E.

